# HIV Rev Assembly on the Rev Response Element (RRE): A Structural Perspective

**DOI:** 10.3390/v7062760

**Published:** 2015-06-12

**Authors:** Jason W. Rausch, Stuart F. J. Le Grice

**Affiliations:** Reverse Transcriptase Biochemistry Section, Basic Research Program, Frederick National Laboratory for Cancer Research, Frederick, MD 21702, USA; E-Mail: rauschj@mail.nih.gov

**Keywords:** HIV, Rev, Rev response element, RRE, Crm1, nuclear export complex

## Abstract

HIV-1 Rev is an ~13 kD accessory protein expressed during the early stage of virus replication. After translation, Rev enters the nucleus and binds the Rev response element (RRE), a ~350 nucleotide, highly structured element embedded in the *env* gene in unspliced and singly spliced viral RNA transcripts. Rev-RNA assemblies subsequently recruit Crm1 and other cellular proteins to form larger complexes that are exported from the nucleus. Once in the cytoplasm, the complexes dissociate and unspliced and singly-spliced viral RNAs are packaged into nascent virions or translated into viral structural proteins and enzymes, respectively. Rev binding to the RRE is a complex process, as multiple copies of the protein assemble on the RNA in a coordinated fashion *via* a series of Rev-Rev and Rev-RNA interactions. Our understanding of the nature of these interactions has been greatly advanced by recent studies using X-ray crystallography, small angle X-ray scattering (SAXS) and single particle electron microscopy as well as biochemical and genetic methodologies. These advances are discussed in detail in this review, along with perspectives on development of antiviral therapies targeting the HIV-1 RRE.

## 1. Introduction

Replication of retroviruses and transposition of endogenous retroelements exploits a unique mechanism of post-transcriptional regulation as a means of exporting their full-length and incompletely-spliced mRNAs (which serve as the genomic RNA and the template for protein synthesis, respectively) to the cytoplasm. This is achieved through the concerted interaction of highly structured *cis*-acting regulatory elements in the RNA genome with a variety of obligate viral and host proteins. Examples of such regulatory RNAs include the constitutive transport element (CTE) of the simian retroviruses (MPMV, SRV-1, SRV-2) [1], the musD transport element (MTE) of murine retroelements (intracisternal A particles and musD) [2], the ~1.7 kb posttranscriptional element (PTE) of gammaretroviruses such as murine leukemia virus [3] and the L1-NXF1 element of human LINE-1 transposons [4]. Studies such as these, which have made a significant contribution to our understanding of cellular processes regulating the fate of RNA, resulted from seminal work performed over 30 years ago to understand nucleocytoplasmic RNA transport in human immunodeficiency virus (HIV). In particular, disrupting the HIV-1 genome in the immediate vicinity of the trans-activator of transcription (tat) open reading frame (ORF) had no effect on its expression levels, but inhibited expression of the gag, pol and env genes, resulting in a severe replication defect [5]. This defect could be corrected by inclusion of an overlapping ORF encoding the regulator of expression of viral proteins, or Rev, a small accessory protein containing both nuclear export and localization signals (NES and NLS, respectively).

**Figure 1:**
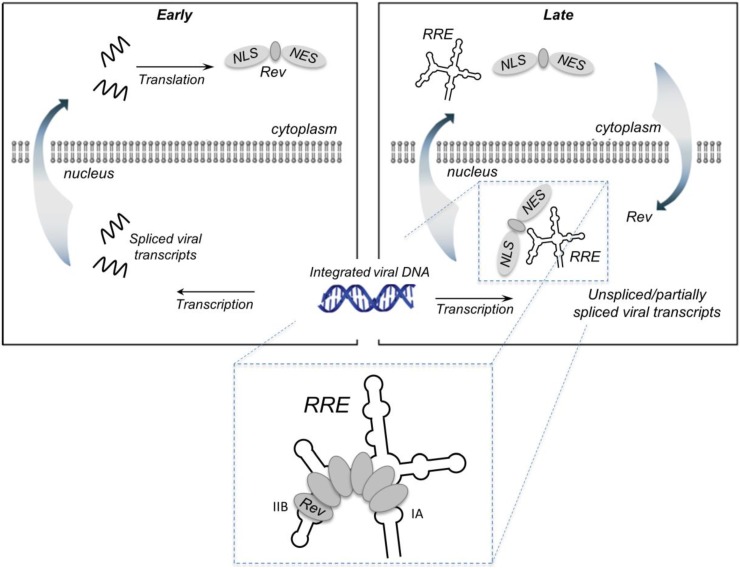
Rev-mediated nucleocytoplasmic transport of HIV-1 RNA containing the Rev RRE. RNA transport: In the early phase of the HIV life cycle, the genomic RNA transcript is completely spliced, generating RRE-free messages, which are transported to the cytoplasm *via* standard nuclear export pathways. One of these messages encodes Rev, which is imported into the nucleus *via* its nuclear localization sequence (NLS). The late phase of the viral life cycle is characterized by the expression of viral proteins encoded by the unspliced (9 kb) or partially spliced (4 kb) RRE-containing mRNAs. These large intron-containing RNAs are retained in the nucleus for splicing/degradation until a sufficient level of Rev accumulates, after which they are exported to the cytoplasm *via* a Rev-dependent export pathway. This involves assembly of the Rev-RRE complex and recruitment of host proteins CRM1 and Ran-GTP *via* the Rev nuclear export sequence (NES). Export of the Rev-RRE -CRM1/RanGTP complex to the cytoplasm provides mRNAs that are translated to produce the remaining viral proteins and full-length genomes that are packaged into the budding virion. Inset: Rev initially binds the IIB secondary structure motif, then cooperatively assembles along the RRE *via* a series of Rev-Rev and Rev-RNA interactions. Rev has also been reported to bind motif IA with high affinity.

The notion that Rev was involved in RNA transport rather than modifying splicing arose from observations of Malim *et al.* [5], who elegantly showed that cytoplasmic expression of the non-spliceable HIV-1 *env* gene was also subject to Rev control. At the same time, these authors identified the Rev response element (RRE), an ~350 nt highly-structured *cis*-acting RNA within the *env* gene (nucleotides 7709–8063), as the target of Rev. The principles of the Rev/RRE axis are outlined schematically in Figure 1. Early in the virus life cycle, Rev and additional HIV regulatory proteins are translated from completely spliced RNAs that are exported from the nucleus in a manner analogous to cellular mRNAs. Subsequently, the arginine-rich NLS facilitates entry of Rev into the nucleus, where it interacts with the RRE in unspliced and incompletely-spliced HIV RNAs. Rev initially binds to stem-loop IIB, a purine rich RNA secondary structure motif (*vide infra*), after which several additional Rev molecules assemble along the RRE to generate the Rev-RRE complex. This complex then recruits Crm1 and other host proteins into a larger complex that is exported from the nucleus into the cytoplasm. The goal of this review is to provide an updated account of our understanding of the HIV Rev-RRE complex with respect to the recently-elucidated structures of the protein and RNA components. For a broader perspective, the reader is referred to excellent reviews from the Cullen [6], Hope [7], Malim [8], Daelemans [9] and Frankel [10] groups.

## 2. Rev Structural Organization and RNA Binding

**Figure 2:**
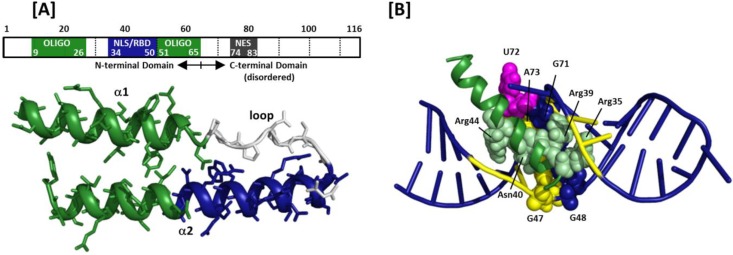
Rev structural organization and arginine-rich motif (ARM)/stem loop IIB complex: (**A**) Rev organization according to primary sequence and 3D structure. The bipartite oligomerization domain and the nuclear localization signal/RNA-binding domain (NLS/RBD) are depicted in green and blue, respectively. The C-terminal domain of Rev, which houses the nuclear export signal (NES), is intrinsically disordered; (**B**) ARM peptide in complex with stem-loop IIB model RNA. The ARM peptide binds in the RNA major groove widened by non-canonical base pairs (G47-A73, G48-G71) and an unpaired, unstacked uridine (U72). These nucleotides are space-filled in the model. Arginines that make specific contacts with nucleic acid bases, and the contacted nucleotides, are highlighted by space-filling and yellow coloration, respectively.

HIV-1 Rev is a 116 amino acid, ~13 kD protein generated by translation of a fully spliced 2 kb mRNA during the early phase of viral replication and organized as shown in Figure 2A [8]. The *N*-terminus of the protein assumes a helix-turn-helix configuration containing two functional domains: the nuclear localization signal and RNA binding domain (NLS/RBD) and the Rev multimerization domain. The NLS/RBD is housed within the distal portion of the helix-turn-helix motif. A stretch of amino acids within α-helix 2 in this domain contains several functionally important arginines and has thus been dubbed the arginine rich motif (ARM). The multimerization domain houses a number of hydrophobic amino acids located at opposite ends of the helix-turn-helix primary sequence, but proximal to each other in three dimensions (3D). Contacts among these hydrophobic residues stabilize the overall Rev structure, and their arrangement generates the two-sided interface for Rev multimerization. Outside of the helix-turn-helix motif, the *C*-terminus of Rev is intrinsically disordered; however, this segment of the protein contains a third, leucine-rich functional domain known as the nuclear export signal (NES). This is the effector domain of Rev, and is required for recruitment of Crm1 and other host proteins that facilitate nuclear export of the Rev-RRE complex.

As its designation suggests, the NLS/RBD of HIV-1 Rev is important for both nuclear import of cytoplasmic Rev and binding to the RRE. Multiple reports indicate that initial Rev binding occurs at a purine rich segment in stem loop IIB, a substructure of the HIV-1 RRE for which Rev has been shown to bind with high affinity (*vide infra*) [11,12,13,14,15]. The first high-resolution picture of the Rev–stem loop IIB interaction was obtained from NMR structures of a Rev ARM peptide in complex with a short synthetic RNA engineered to stably recapitulate the IIB RNA substructure [16]. The averaged structure of the ARM-IIB complex is depicted in Figure 2B, wherein nucleotide numbering reflects that used for the artificial IIB construct in the file deposited in the Protein Data Bank (PDB). The IIB binding site is notable in that it contains two non-canonical base-pairs (G47-A73 and G48-G71) separated by an unstacked, bulged uridine (U72) [17,18]. This arrangement serves to widen the helical major groove at the site where the Rev peptide contacts the RNA [16,19,20]. Upon binding, the Rev ARM penetrates deeply into the stem loop IIB major groove [16,21,22,23], thereby inducing a conformational change that widens the groove even further [24,25,26]. Four ARM residues make base-specific contacts with nucleotides in stem loop IIB: Arg35 and Arg39 contact nucleotides U66, G67 and G70 on one side of the RNA major groove, while Asn40 and Arg44 contact U45, G46, G47 and A73 on the other. In addition, Thr34, located at the non-helical turn segment of the Rev helix-turn-helix motif, and six arginine residues (Arg38, Arg41–Arg43, Arg46 and Arg48) within the ARM, interact nonspecifically with the RNA sugar-phosphate backbone.

More recently, a crystallographic structure of a rev dimer in complex with an engineered RRE-like RNA was resolved that has greatly enhanced our understanding of both Rev interactions and how Rev binds RNA [27]. Unlike the NMR structure, the 47-nt RNA in the Rev dimer complex contains both the IIB binding site and an adjacent “junction site” engineered to resemble the three-way junction within stem loop II. This secondary site contains a non-canonical G-A base pair like that found in IIB, as well as an unpaired U. The unpaired U and adjacent A were shown to be essential for binding of the second Rev, as their removal results in a complex in which only a single Rev is bound. In the crystal structure, the ARMs of the dimerized Rev molecules bind along the RNA major grooves of the adjacent binding sites. Contacts at the IIB site closely resemble those observed in the NMR structure, with the notable exception of those involving Rev amino acid Asn40. At the junction site, all contacts except those involving Arg43 and Arg44 are with the RNA phosphate backbone, consistent with finding that binding at the second site is relatively sequence non-specific and requires only that the site contain an RNA bulge [28]. While the rotational positioning of the two Rev ARMs in the major grooves of the IIB and junctional binding sites appears to be similar, their linear positioning is displaced by approximately the length of an alpha helical turn. The structure also addresses how binding of the Rev dimer to adjacent sites on the RRE RNA affects the Rev dimer interface, which will be discussed below.

Another Rev binding site in RRE stem loop I has been identified using structural and biochemical techniques [29]. Like the IIB and junctional sites, the stem I site (designated site IA) is also comprised of a purine-rich bulge in which the major groove might be expected to be widened, flexible, and/or defined by non-canonical G-A base pairs. However, mutational analysis suggests that residues Arg38, Arg 41 and Arg 46 are crucial for binding at site IA, indicating a different rotational positioning of Rev in the RNA major groove and suggesting that the Rev-RRE interface is flexible and may be substantially different at distinct binding sites. Other segments of stem I have been implicated in Rev binding [30], but the Rev-RNA interactions at these sites have not been characterized structurally.

## 3. Structural Basis for Rev Oligomerization

Although oligomerization has been shown to be an important aspect of Rev function in virus replication [29,31,32], this property was first observed with recombinant protein. At low concentrations, Rev exists as monomer [11], dimer [33] or tetramer [11,34]. Above a critical concentration of ~80 ng/mL (~6 µM), however, Rev forms regular, unbranched filaments of indeterminate length [11,33,35,36]. The structural basis for filament formation resides in the two-sided multimerization interface in which a given Rev molecule can be flanked by additional Rev on either side.

The oligomerization domain of HIV-1 Rev is a flat, two-sided structure formed by juxtaposition of two α helical regions located at opposite ends of the Rev helix-turn-helix motif primary sequence. The domain has a polarity named for the sides of a coin, with the “heads” (H) and “tails” (T) faces interacting specifically with their equivalent counterparts in adjacent Rev molecules; *i.e.*, “H/H” or “T/T” interfaces are preferentially formed in Rev oligomers [37]. Although the T/T Rev dimer is more stable than the H/H and is the form assumed by Rev dimers in solution and after initial binding to the RRE IIB substructure, a high resolution structure of a Rev dimer containing an H/H interface was the first to be resolved by X-ray crystallography [38]. Crystal formation was promoted and filament formation inhibited in this case by blocking the outer T faces of the Rev dimer with monoclonal F_ab_ fragments. Although present in the crystalized protein, the disordered C-terminal portion of Rev, including the NES, was unresolved.

**Figure 3:**
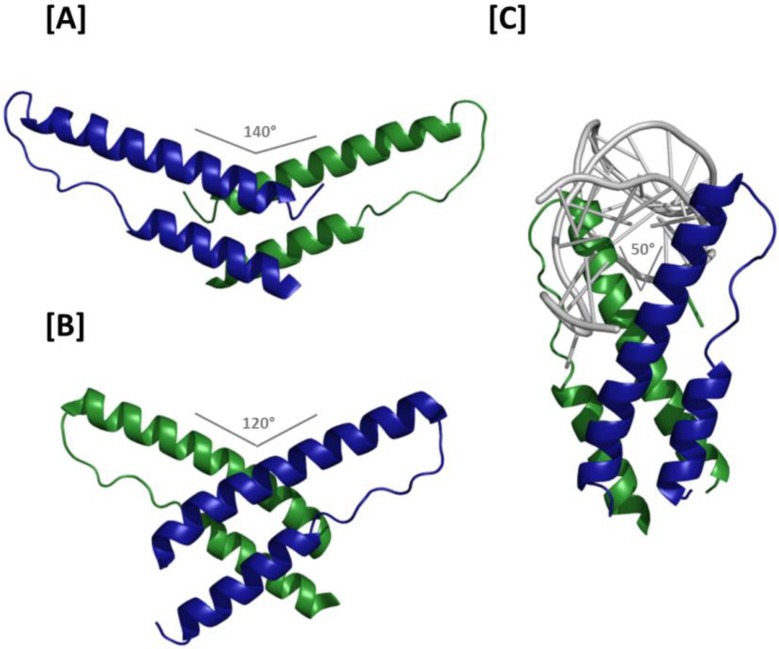
Crystal structures of Rev dimers: (**A**) Ribbon representation of the H/H Rev dimer. The angle formed by the two ARMs is 140°, which separates the apices of the two NLS/RBDs by approximately 8 nm and precludes binding at adjacent sites on an RNA helix; (**B**) T/T Rev dimer structure. In the absence of RNA, the ARM angle is 120°. Binding at adjacent RNA sites is conceivable, but higher order Rev multimerization would likely require a reduced ARM angle at H/H interfaces; (**C**) Structure of the T/T Rev dimer in complex with RNA. The two ARMs bind RNA at adjacent sites on the helix and are oriented at an angle of 50° relative to each other. The Rev oligomerization and RNA helical axes in this structure are roughly parallel, thereby facilitating consecutive binding and the higher order structures proposed in the jellyfish model of Rev assembly.

In support of prior genetic and NMR structural data [16,37], a hydrophobic core comprising residues Leu12, Ile19, Leu22, Tyr23 of alpha-helix 1 and Trp45, Ile52, Ile59, Leu60 and Tyr63 of α-helix 2 were shown to be major contributors to Rev helical hairpin stability. Intermolecular interactions among an overlapping set of multimerization domain hydrophobic residues (Leu12, Leu13, Val16, Ile19, Leu60 and Leu64) were shown to comprise much of the H/H interface, of which the roles of Leu12, Val16 and Leu60 were previously established genetically [37]. Because the H/H junction involves residues located at the extreme pronged end of the Rev helix-turn-helix, viewing the structured portion of the dimer from the intermolecular axis orthogonal to the plane of the interface demonstrates that the two molecules assume a “V-like” configuration relative to each other (*i.e.*, rather than one resembling an “X”) (Figure 3A). Moreover, the helix-turn-helix motifs are arranged such that the two ARMs form an angle of approximately 140°. The spacing between NLS/RBDs and the opposing trajectories of the two motifs place their extreme termini approximately 8 nm apart, suggesting that the ARMs in such a dimer would be unlikely to bind the RRE in adjacent regions of the major groove on the same helix. Instead, a model based on superimposition of H/H Rev dimer crystal structure and the ARM/IIB NMR structure that suggests the H/H dimer may be best suited for linking either two separate RNAs or two RNA helices located in distant regions of the same RNA [38].

Details of the T/T Rev dimer interface were first resolved in a crystal structure in which H-surface residues Leu12 and Leu60 were mutated to suppress higher order multimerization [39] (Figure 3B). Despite these modifications, asymmetric units in these crystals contained four Rev molecules linked sequentially by T/T, H/H and T/T interfaces, where the H/H interaction matched that observed in the original dimer crystal structure almost exactly. Packing of hydrophobic residues on the T surfaces of adjacent Rev molecules formed an interface that buries over 1500 Å^2^ of surface area. Leu18 and Ile55 form symmetric contacts between monomers at the T/T interface, are highly conserved, and essential for cooperative RNA binding and export [29,37,40]. Phe21, Leu22 and Ile59 are likewise present at the dimer interface, although these residues are also important for stabilizing monomeric Rev. From the vantage point of the oligomerization axis, the ordered portion of the T/T Rev dimer assumes a configuration resembling an asymmetric “X”, with long and short NLS/RBD and multimerization domain protrusions flanking the Rev-Rev interface on either side, respectively. Moreover, at 120°, the ARM angle formed in the T/T dimer is narrower than observed in the H/H dimer structure. A molecular model of the T/T dimer in complex with a short IIB-like RNA suggests that both opposing ARMs could bind in the major groove on the same face of an RNA helix with contacts separated by approximately one A-form helical turn. It is further suggested that Rev-Rev and Rev-RNA interactions may propagate from the IIB initiation site along a contiguous region of the RRE, placing the Rev molecules—each with a disordered NES—projecting away from the RNA in a common direction like “tentacles of a jellyfish” [39]. The jellyfish model provides important insight into how multiple Revs may assemble on a comparatively large RRE, and will be discussed in more detail below. However, it is worth noting that in this initial manifestation of the model, the helical axis of the RNA was nearly perpendicular to the axis of Rev oligomerization—an arrangement that would likely be incompatible with the proposed coordination of multiple Revs binding at adjacent sites on the RRE.

How Rev-Rev and Rev-RNA interactions can occur concomitantly was largely explained by the aforementioned co-crystal structure in which a T/T Rev dimer binds a truncated RRE RNA at adjacent IIB and junctional sites [27]. ARM binding in the RNA major groove at the two sites appears to change the organization of the T/T interface relative to the naked Rev dimer, and a reciprocal effect can be observed in the distorted binding site major grooves. Among of the more important consequences of this reorganization are that the angle formed by the two Rev ARMs is reduced to ~50° and the Rev multimerization and RNA helical axes are nearly parallel (Figure 3C). Both of these observations are compatible with the jellyfish model of Rev assembly.

Relative to the naked dimer, the T/T interface of the Rev dimer-RNA complex is rotated around Ile55, substantially altering contacts among the hydrophobic residues of both T-surfaces. Although the interacting residues at the T/T interface are largely the same regardless of whether RNA is present, specific points of contact are altered considerably. Collectively, surface area buried at the T/T interface in the Rev dimer-RNA complex is reduced by ~33% (to ~1000 Å^2^), suggesting that the dimer may be less stable in the presence of RNA. However, the energetic favorability of RNA binding likely compensates for reduced hydrophobic interactions, thereby rendering the Rev-dimer-RNA complex more stable than the naked dimer. The Phe21 residues that facilitated dimerization in the naked dimer are excluded from the T/T interface in the Rev-dimer RNA complex; moreover, reciprocal Gln51-Gln51 hydrogen bonding is observed only in the presence of RNA. Interestingly, introducing a Gln51Ala mutation into Rev appears to both impair Rev dimerization and reduce the affinity of Rev for the truncated RRE RNA approximately 30-fold. However, the effects of this mutation on the fully assembled Rev-RRE complex are relatively modest, suggesting that Gln51 hydrogen bonding may not be as important during later stages of Rev assembly.

## 4. Secondary and 3D Structures of the RRE

Our structural understanding of Rev, Rev dimer and Rev–IIB interactions has been greatly facilitated by X-ray crystallography and NMR. Using the same approaches to study the structure of the HIV-1 RRE is problematic, however, given the size and flexibility of this highly structured RNA element. For the most part, experimental approaches have been restricted to using a number of enzymatic and chemical probing techniques to characterize the RRE secondary structure. Although these efforts sometimes produced differing secondary structural models for similar RRE variants, common structural features have been identified that help both in developing 3D models of the motif and for understanding how Rev assembles along this conserved segment of RNA.

**Figure 4:**
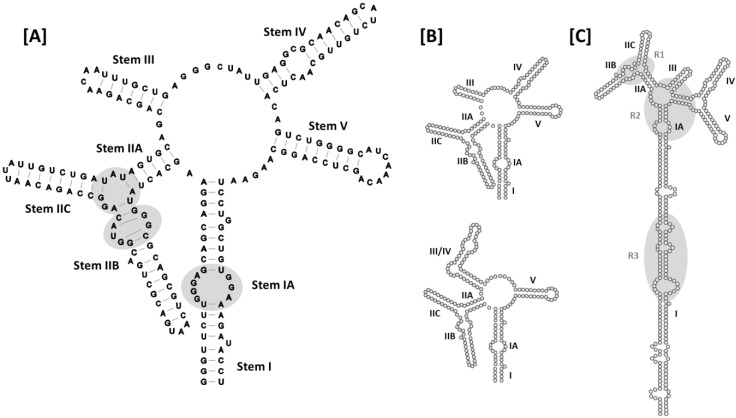
Secondary structures of HIV-1 RREs: (**A**) 5-stem structure. Sub-structure designations and base-pairing patterns, including non-canonical G-A and G-G base pairs in stem IIB, are indicated. Established Rev binding sites at IIB, the stem loop II junction and stem IA are also shown (gray ovals); (**B**) Comparison of the 5- and 4-stem structures. Differences between the two models are limited to the base pairing patterns of nucleotides comprising stem loops III and IV, and stem loop III/IV, in the respective structures. Due to space limitations, stem I has been truncated in the models presented in panels (**A**–**C**). Alternative 5-stem RRE structure proposed for an ARV-2/SF2 HIV-1 isolate. The entire RRE is shown. Stem I is truncated relative to the original 5-stem model, and there are more single-stranded nucleotides separating stem I and stem IIA. In addition, base pairing across the central junction separates stem loops IV and V from the rest of the alternative 5-stem structure. R1, R2 and R3 refer to Rev binding regions 1–3 identified for this RRE variant by time-resolved selective 2′-hydroxyl acylation analyzed by primer extension (SHAPE).

Although the HIV-1 RRE is approximately 350 nt in length, the secondary structure of this RNA element was initially characterized using RNA folding prediction software together with enzymatic and chemical RNA probing experiments conducted using a truncated (~235 nt), *in vitro* transcribed version of the RNA [41]. The sub-structure designations defined in this seminal work will also be used here. In the original model, the HIV-1 RRE RNA assumes a secondary structure comprised of five stems, stem loops or bifurcated stem-loops (I-V) arranged around a central 5-way junction (Figure 4A). Stem I is the longest of the stems/stem-loops, and is interrupted by a number of internal loops and bulges of varying size. Some of these internal loops are purine rich, and that most proximal to the central junction (stem IA) has been identified as a high-affinity Rev binding site [29]. Stem-loop II is bifurcated to form smaller substructures designated IIA, IIB and IIC. As previously noted, IIB contains a purine-rich internal segment characterized by non-canonical G-A and G-G base pairing and a widened major groove, and has been identified as the site at which Rev initially binds to the RRE to initiate assembly [31,32,42]. Moreover, according to the Rev dimer-RNA co-crystal structure [27], the adjacent stem-loop II junctional region serves as the binding site for the second Rev. Proceeding clockwise around the central junction in Figure 4A, stem loops III-V complete this 5-stem secondary structural model of the RRE.

A later 4-stem model obtained using enzymatic and chemical probing techniques suggests that stem loops III and IV, together with the intervening loop region, are organized to form a hybrid III/IV stem loop [32] (Figure 4B). While the central junction is compacted somewhat in this alternative folding, no additional differences between the 4- and 5-stem RRE secondary structures were proposed. Which of the two structures is assumed by the RRE in the context of HIV replication has been a subject of considerable debate, with multiple studies supporting both forms [2,43,44]. Recently, however, it has been demonstrated using native gel electrophoresis and in-gel SHAPE [45] that the HIV-1 RRE can assume either the 5- or the 4-stem conformation [46]. Moreover, supporting virological data suggest that the two forms may not be equivalent, *i.e.*, HIV-1 housing a mutant RRE that exclusively assumes the 5-stem conformation outgrows virus with a 4-stem RRE in growth competition experiments. This finding is in agreement with the observation that RRE61, an RRE mutant shown to assume a conformation resembling the 5-stem structure, confers resistance to the *trans*-dominant Rev mutant RevM10 [2].

Recently, another secondary structural model was proposed for the RRE of an ARV-2/SF2 HIV-1 isolate (Figure 4C) that closely resembles the RRE configurations proposed for HIV-2 and SIVmac [30,43,47]. This model resembles the original 5-stem HIV-1 RRE structure in that stem-loops II, III, IV and V are identical in the two versions. However, in the more recent model, the loop region between I and IIA is larger than in original model, with a 6-basepair “bridge” across the central junction separating stem loops IV and V from the rest of the structure. Since nucleotides contributing to these alternative arrangements are base-paired at the proximal terminus of stem I in the original structure, the stem I motif is four base pairs shorter in the newer model.

The modified 5-stem RRE structure is arguably the most compatible with the jellyfish model of Rev assembly, as single-stranded, purine rich, Rev binding sites at the IIB/stem II junction, central junction and proximal stem I internal loop are more evenly spaced than in other secondary structural models. However, the authors of this study also provide evidence for a heretofore unrecognized Rev binding region among the purine-rich internal loops located near the center of stem I, and propose a novel mechanism of Rev assembly that differs somewhat from the jellyfish model to explain binding at that site [30]. This model of assembly will be discussed in more detail in the following section.

While varying RRE sequence and secondary structure has been shown to affect RRE function, the nature and degree of these effects are not entirely predictable. For example, whereas large engineered changes such as deletion of stem loop II almost completely abolishes Rev binding in *in vitro* assays [14], disruption of stem loops III and IV (or III/IV) does not. The latter changes do, however, substantially impair Rev/RRE-mediated nuclear export function in cell culture [46]. Nuclear export of HIV-1 RNA is similarly reduced upon serial truncation of RRE stem I, although this process is gradual, and considerable function is retained in RRE variants as small as ~230 nt [32].

The effects of natural RRE sequence and structural variations in the context of viral replication can be more difficult to interpret. Since the RRE is embedded in the *env* gene, changes in RRE sequence may affect the functionality of Env as well as the nuclear export of HIV-1 RNA. Another consequence of this dual functionality may be that genetic flexibility is limited, making the RRE sequence a potentially promising target for antiviral therapies. Despite these seeming limitations, RRE sequences derived from clinical samples do exhibit a degree of genetic variation, and the effects of these sequence differences are not always easy to explain. For example, in one study, the function of patient-derived RRE variants appeared to be more affected by select single nucleotide polymorphisms than by more pronounced changes predicted to substantially alter RRE secondary structure [48]. In other work, select polymorphic RREs obtained from clinical isolates were shown to decrease Rev-dependent nuclear export 2–3-fold, although a corresponding effect on RRE structure was not established [49]. Although it is not clear how specific RRE mutations correlate with RRE structure and function, it has been suggested that mutational attenuation of RRE function could potentially serve as a natural means of down-regulating HIV-1 replication during the course of infection [46].

**Figure 5:**
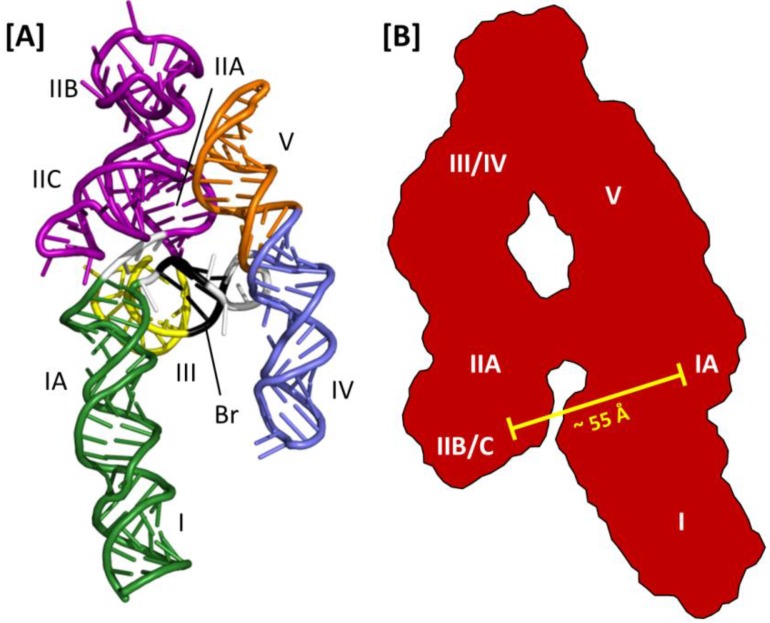
Three-dimensional models of the HIV-2 and HIV-1 RREs: (**A**) HIV-2 RRE model structure obtained using SHAPE and molecular modeling with RNA Composer. Substructures homologous to those reported for the HIV-1 RRE are indicated. The region of base pairing that bridges the gap between stem loops IV and V and the rest of the structure is also shown (Br, black ribbon); (**B**) A-like SAXS envelope obtained for a truncated HIV-1 RRE (233 nt). Sub-structure designations and positioning are determined by fitting an RRE molecular model into the SAXS envelope. The molecular model was generated using the RRE 4-stem secondary structure as a template. High-affinity Rev binding sites at IIB and IA are separated by approximately 55 Å.

Thus far, molecular modeling and small angle X-ray scattering (SAXS) have been the only means of generating 3D models of the RRE. One option for the former approach is to use RNA Composer, a web-based RNA folding application that uses homology modeling and energy minimization to assemble a 3D RNA structure from primary sequence and an associated secondary structure map [50]. This software was used to generate the 3D model of the HIV-2 RRE depicted in Figure 5A [47]. While less is known about HIV-2 Rev assembly on its cognate RRE, alignment of the homologs of IIB, the central junction and stem loop I in this model suggest that the RRE 3D structures and mechanisms of Rev assembly may be similar between HIV-2 and HIV-1.

A combination of SAXS and molecular modeling was used to generate a 3D model of the HIV-1 RRE [51]. Using the 4-stem secondary structure map as a model, individual sub-structures were constructed (e.g., stem loop III/IV) and analyzed by SAXS. The molecular boundaries of the sub-structures were then mapped onto the global envelope of a truncated (233 nt) HIV-1 RRE, which was itself used to constrain and define the 3D organization of the RNA predicted by molecular modeling (Figure 5B). In the resulting structure, the HIV-1 RRE assumes an A-like configuration in which stem IIA and stem-loop III/IV are collinear, stem I is collinear with stem-loop V and the two extended motifs are centrally linked by a rigid, elongated 4-way junction. Moreover, whereas the loop regions of III/IV and V are proximally located, and perhaps contacting each other at the apex of the A-like configuration, IIB and the putative high affinity Rev binding site in stem I are opposite each other and separated by ~55 Å. Because this distance approximates the separation between the ARMs of the Rev dimer in the absence of RNA [39], it was suggested that dimeric Rev spans from IIB to the high affinity site in I in the early stages of assembly [51]. In this model, multimerization then proceeds outward from the initiating dimer in both directions along the multimerization axis on one face of the A-like RRE RNA.

Subsequent SAXS analysis of the full length RRE extends the preceding model to include all of stem I [30]. These data suggest that an intact stem I folds back on itself so that the distal portion of the motif interacts with the proximal portion, and perhaps also with regions near the central junction. Interestingly, although A-like RRE SAXS envelopes are reported in both studies, probably in part because the former model was used to derive the latter, the 4-stem secondary structure used to model the former structure was not used in the more recent study. Instead, SHAPE analysis [52,53] determined that the RRE variant used in the latter work assumed the modified 5-stem structure with base-pairing across the central junction. While this secondary structural variation was used for subsequent SHAPE-based mapping of Rev binding sites on the RRE, the modified 5-stem RRE was not modeled into the A-like SAXS envelope in this study.

## 5. Rev Assembly on the RRE

It is well established that multiple copies of HIV-1 Rev bind the RRE, and when this capacity is mutationally abolished, Rev-mediated nuclear export is adversely affected [32,42,54,55]. The stoichiometry of the saturated Rev-RRE complex, however, remains a subject of some debate. Reported binding ratios range from of 5:1 to 13:1 Rev/RRE, with an 8:1 ratio most often represented in early work in this area [11,32,33,39,56,57,58,59,60,61,62,63]. A study of particular note utilized surface plasmon resonance and a 244-nt RRE to measure Rev binding kinetics, with results indicating that up to 10 Rev molecules could bind the RRE before the complex became saturated [63]. Moreover, while the kinetics of the first four binding events suggest that Rev binding is sequential and specific, the subsequent Rev binding appeared to occur non-specifically. Most recently, a Rev/RRE ratio of 6:1 was determined for a 242-nt RRE using size exclusion chromatography [39]. The hexameric-Rev-RRE in this study was also shown to migrate as a discrete species by native polyacrylamide gel electrophoresis, suggesting structural uniformity, while complexes containing the Rev Leu18Gln/Leu60Arg multimerization mutant migrated as a broad, diffuse band to a position consistent with a Rev/RRE ratio of approximately 3–4:1.

Biochemical and biophysical experiments, together with single complex FRET measurements, demonstrate that Rev assembles on the RRE one molecule at a time [2,64], binding initiates at stem-loop IIB [11,12,14,15,31] and the binding of successive Rev molecules is cooperative [29]. Nuclease protection analysis further indicates that Rev binding occurs principally on stem loop IIB and stem I of the RRE, as other structural motifs remained susceptible to nuclease cleavage in the presence of Rev [32]. Although a great deal is now known about how Rev interacts with itself and with RNA, the precise sequence of events required for Rev assembly, the positioning of individual Rev molecules on the RNA and the 3D structure of the saturated Rev-RRE complex remain unknown.

**Figure 6:**
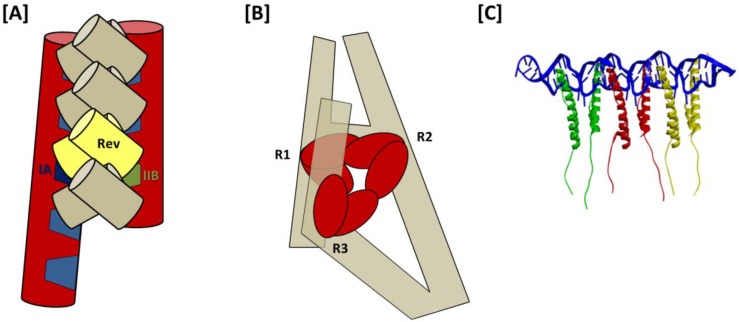
Models for assembly of HIV-1 Rev on the RRE: (**A**) “Bridging” model. The first two Rev in the complex bind to IIB and IA high affinity sites on the A-like RRE, as well as to each other, thereby forming a Rev dimer that “bridges” the distance separating two sub-structures. This proposal is based largely upon the observations that the distances separating the apices of the Rev arms in the T/T dimer crystal structure and the IIB and IA motifs modeled to fit the HIV-1 RRE SAXS envelope are both approximately 55 Å. After this initial Rev dimer-RRE complex is formed, assembly is proposed to propagate in both directions along the A-like RRE structure and involve stem loops III/IV, V and more distal portions of stem I; (**B**) Stem I “loop-back” model. Based on analyses using SAXS and time resolved SHAPE, it has been proposed that following relatively rapid assembly of Rev dimers at R1 and R2 (R1, Region 1—IIB and stem II junction; R2, Region 2—central junction and IA), the remaining dimer in the hexameric Rev complex assembles at R3 (Region 3—a series of purine-rich bulges in a more distant segment of stem I). RNA tertiary interactions bring the stem I terminus into proximity with the RRE central junction (even in the absence of Rev), and the last Rev dimer is accommodated in the fully assembled complex by an induced fit mechanism of conformational sampling; (**C**) Jellyfish model. Rev initially binds to the IIB high-affinity site, after which five additional Rev molecules assemble on the RRE *via* a series of consecutive T/T and H/H multimerization domain interactions. These Rev-Rev interactions are facilitated by concomitant Rev ARM binding at adjacent regions on the RRE RNA, such as is observed in the T/T Rev dimer-RNA crystal structure. The six *C*-terminal domains in this putative Rev-RRE complex would be expected to project in a common direction, which would in turn facilitate NES binding at the Crm1 dimer interface and promote nuclear export.

From the 3D structure of a 232-nt RRE obtained using molecular modeling in conjunction with SAXS, a model for Rev assembly was proposed in which 8 Rev molecules are coaxially arranged along one face of the A-like structure of the truncated RRE (Figure 6A) [51]. Support from this proposal comes primarily from observations that the high affinity Rev binding sites on IIB and stem-loop I are separated by ~55 Å, matching the approximate separation between distal portions of the ARMs in the T/T Rev dimer crystal structure [39]. However, the model also suggests direct binding of Rev to stem loops III/IV and V in higher order complexes and does not involve distal regions of stem I in Rev assembly. Both of these suppositions are inconsistent with prior nuclease protection analysis [32]. The high resolution Rev-dimer-RNA co-crystal structure [27], in which tandem NLS/RBSs bind at adjacent sites on the same helix likewise does not support the IIB-I bridging model of Rev assembly suggested by Wang and colleagues.

As noted previously, SAXS analysis of the full-length RRE suggests that the distal segment of stem I folds back on itself over one face of the A-like RRE structure [30]. Probing experiments further show highly variable sensitivity to chemical acylation at select regions of the RRE in the presence and absence of Rev. These area are designated Regions 1–3, and correspond, respectively, to (i) IIB and the stem loop II junction; (ii) the central junction and proximal purine rich internal loop of stem I; and (iii) a sequence of three adjacent, purine rich bulges located near the center of stem I. These regions of variable acylation sensitivity reportedly mark Rev binding sites, with Region 1 containing sites included in the Rev-dimer-RNA crystal structure [27].

Based on these data, a model for Rev assembly was presented in which Rev dimers bind sequentially at Regions 1–3 (Figure 6B). As in both previous models, assembly is proposed to initiate at the IIB nucleation site of Region 1. Moreover, as would be predicted by the jellyfish model, Rev binding at Regions 1 and 2 is temporally coupled, likely due to their spatial proximity. Unlike in the jellyfish model, however, this intriguing new model suggests that the 4-Rev complex subsequently undergoes an induced-fit conformational change to accommodate a third Rev dimer binding at Region 3, which is not adjacent to Regions 1 and 2 in the RNA secondary structure but is brought into proximity by tertiary RNA interactions. Specific Rev-Rev associations within the hexameric complex are not otherwise detailed in this model, and the relative positioning and orientation of individual Rev molecules in the fully assembled complex is likewise not predicted. Moreover, it is worth noting that in seeming contradiction to this model, a hexameric Rev-RRE complex has been reported to assemble on a 242-nt truncated RRE that lacks almost all of the Domain 3 Rev binding site(s) [29].

In the jellyfish model, Rev assembly extends from the IIB nucleation site through the RRE central junction and into the proximal segment of stem I, including stem IA (Figure 6C). Size exclusion chromatography suggests that coordinated assembly stops after six Rev molecules have been bound in the complex [29]. Rev-Rev and Rev-RNA interactions in such a complex would be consistent with the recent Rev-dimer RNA crystal structure [27], but a stable Rev hexamer would likely require similarly flexible H/H interface. More specifically, it would be necessary that the angles formed by the ARMs in adjacent Rev molecules are relatively narrow at both T/T and H/H interfaces, allowing for sequential binding at widened major grooves in adjacent segments of a contiguous RNA helix. Such an arrangement would be consistent with nuclease protection studies that suggest that Rev binds at IIB and along stem I [32], and also predict that the Rev effector domains align along the same face of the RRE.

Some RRE mutations have been shown to alter both RNA secondary structure and the capacity of the RRE to mediate nuclear export of HIV-1 RNA, yet do not appear to affect the binding kinetics or stoichiometry of Rev binding [2,46]. Such observations may be explained by aberrant Rev-RRE complexes that assemble with normal kinetics but where the relative orientations of Rev molecules are affected. Such subtle distinctions among complexes would be invisible in most biochemical assays, yet may substantially affect Rev-RRE function in a cellular context. In a recent study, the structure of a nuclear export complex containing Rev, the RRE and a Crm1 dimer was elucidated using single particle electron microscopy [65]. In this elegant work, the authors demonstrate that the Rev-RRE complex binds at the Crm1 dimer interface. It is suggested that each Crm1 subunit is bound by three NES domains of the hexameric Rev, although there is sufficient diversity among the complexes to allow for other binding stoichiometries. Crm1 binding appears to require a functional NES, as Rev bearing a dominant-negative M10 mutation [66] in the NES did not bind Crm1 under any condition. Although the resolution of these images is insufficient to definitively verify the jellyfish arrangement of Rev on the RRE, the observed interactions are consistent with a hexameric Rev-RRE in which the NES domains are concentrated in a small area and oriented in a common direction.

## 6. Therapeutic Targeting of the HIV-1 RRE

Based on its crucial role in HIV replication, interrupting RRE function clearly offers an attractive avenue for therapeutic intervention. The notion of using a transdominant negative version of the HIV-1 Rev protein harboring mutations in its nuclear export signal (RevM10), while disrupting export of Rev-dependent viral transcripts [67], was later shown to promote acquisition of resistance-conferring mutations [2]. Early attempts to target the HIV-1 RRE with small molecules have demonstrated the usefulness of neomycin B and related aminoglycosides as structural probes [68,69]. However, the therapeutic potential of this class of antagonist faces the challenges of specificity and poor cellular uptake [70]. Thus, alternative approaches to target the Rev/RRE interaction are warranted.

Both structural and mutagenic analyses have shown that the α-helical arginine-rich motif (ARM) of Rev mediates a critical interaction with the RRE by inserting into the major groove of an asymmetric bulge in IIB. Based on these observations, Mills *et al.* [71] synthesized a series of ARM-like conformationally-constrained (*i.e.*, α-helical) peptidomimetics to antagonize the Rev/RRE interaction. Of 15 candidate peptidomimetics examined, one bound the RRE with an affinity equivalent to that of the Rev ARM (Kd ~50 nM), while control experiments indicated that its unconstrained counterpart showed little specificity. An alternative approach has suggested targeting the RRE with an ARM peptide modified to incorporate reactive metal chelates [72,73]. This strategy envisions “catalytic” metallo-inhibitors, which, following destruction of their target biomolecule, would dissociate and circulate through the viral RNA population, and thus would not be required in saturating amounts to achieve maximum potency. Bifunctional metallo-inhibitors linking a high affinity targeting motif (the Rev ARM) with a metal chelate complex capable of damaging RNA in its vicinity *via* a variety of oxidative chemistries have been synthesized, and proof of concept was demonstrated by the ability of Cu^2+^-Gly-Gly-His-ARM complexes to selectively cleave RRE RNA *in vitro* [74] and *in vivo* [72]. An extension of this strategy has investigated the oxidative properties of Rev ARM peptides linked to metal chelators such as tetraazacyclododecane-tetraacetic acid (DOTA), diethylenetriamineepentaacetic acid (DTPA), ethylenediaminetetraacetic acid (EDTA) and nitrilotriacetic acid (NTA) [75]. All complexes retained high affinity binding to RRE IIB (0.2–16 nM) and their Cu^2+^-bound chelates efficiently induced RRE cleavage, with activity varying in the order Cu-NTA-Rev > Cu-DOTA-Rev > Cu-DTPA-Rev > Cu-EDTA-Rev. From a therapeutic perspective, oxidative damage of the RRE would result in dissociation of the metal chelate-ARM complex, which can be “reactivated” by reducing agents such as ascorbic acid or glutathione, whose concentrations are sufficiently high *in vivo.* Also, since the metal is extremely tightly chelated (Kd ~10^−15^ M), the risk of toxicity due to metal ion leaching from the complex would be negligible.

Following initial reports that promoted multiple antigenic peptides for vaccine design [76], branched peptides comprising natural or unnatural amino acids, or combinations thereof, have been gaining attention as therapeutic agents, based on their potential for multivalent targeting of RNA and their resistance to proteolysis [77]. In screening a branched peptide boronic acid (BPBA) library whose boronic acid substituent was designed to mimic an acceptor for RNA 2′ OH groups and improve selectivity for RNA over DNA, Zhang *et al.* [78] identified BPBA1, a compound that bound RRE IIB with micromolar affinity and a 1:1 stoichiometry. Mutational studies supported the notion that the IIB tertiary structure was necessary for high affinity binding of BPBA1, while enzymatic footprinting highlighted several nuclease-insensitive regions in the presence of the branched peptide. Finally, studies with a fluorescent BPBA1 derivative suggest cellular uptake can be achieved, although suppression of HIV-1 replication remains to be established.

Of the currently available approaches, small molecules have superior delivery properties and can be “fine-tuned” through medicinal chemistry. With respect to the Rev/RRE interaction, several recent studies support their further development. By combining NMR spectroscopy and computational molecular dynamics, Steizer *et al.* have taken advantage of virtual screening to identify several compounds that antagonize the Tat/TAR interaction by interacting with nucleotides of both the apical loop and trinucleotide bulge [79]. Our laboratory has exploited small molecule microarrays (SMMs) with fluorescently-labeled, structured RNA motifs, where we identified a novel chemotype that binds the TAR hairpin with micromolar affinity, inhibits virus replication in culture and is not cytotoxic [80]. Finally, Informa, a computational approach to designing lead small molecules targeting RNA motifs based on sequence alone, has been used to identify a bioactive benzimidazole that induces apoptosis in cancer cells by sequestering the nuclease processing site of pre-micro RNA-96 [81]. Extending this approach to target the RRE would seem a logical next step.

Although this section has concentrated on small molecule inhibition of the Rev/RRE axis by targeting the viral *cis*-acting RNA, we should not rule out the possibility of targeting the HIV Rev protein to antagonize its interaction with host factors, thereby interrupting nucleocytoplasmic transport of unspliced and singly-spliced viral RNAs. In this respect, Campos *et al*. [82] have recently made the exciting discovery that the quinolin-2-amine ABX464 specifically targets Rev-dependent nuclear transport of HIV RNA without compromising cellular function. The lack of toxicity displayed by ABX464, combined with its ability to suppress viral load sustainably after treatment arrest, represent another powerful addition to the armament of HIV antivirals that will be needed until either a cross-clade vaccine or functional cure is achieved.

## 7. Summary and Perspective

The Rev-RRE interaction is vital to HIV replication and therefore constitutes an important axis for antiviral therapy. Although recent SAXS and crystallographic studies have greatly enhanced our understanding of these two viral components on a structural level, much remains to be learned—particularly regarding the details of Rev assembly and the overall structure of the nuclear export complex. Biochemical studies tell us that six Rev molecules cooperatively assemble on each RRE, and the resulting complex binds Crm1 at the dimer interface according to single particle electron microscopy. The latter study also suggests that while they share many common features, the structures of Rev_6_-RRE-Crm1_2_ complexes may not be completely uniform. Other factors, including the observed flexibility in the Rev T/T interface, potential flexibility in the H/H interface, and variability in RRE RNA sequence and secondary structure suggest this as well. It is conceivable, therefore, that select components of the “bridging”, “loop-back” and jellyfish models of Rev assembly may all contribute to formation of the nuclear export complex in the context of the HIV-infected cell.
